# Sex-based differences in left ventricular remodeling in patients with chronic aortic regurgitation: a multi-modality study

**DOI:** 10.1186/s12968-022-00845-5

**Published:** 2022-02-22

**Authors:** Albree Tower-Rader, Isadora Sande Mathias, Nancy A. Obuchowski, Duygu Kocyigit, Yash Kumar, Eoin Donnellan, Michael Bolen, Dermot Phelan, Scott Flamm, Brian Griffin, Leslie Cho, Lars G. Svensson, Gosta Pettersson, Zoran Popovic, Deborah Kwon

**Affiliations:** 1 Department of Cardiology, Harvard Medical School, Massachusetts General Hospital, 55 Fruit St, Yawkey 5B, Boston, MA 02114 USA; 2 Department of Cardiology, Houston Methodist Hospital, Weill Cornell Medical College, 6565 Fannin St., Houston, TX 77030 USA; 3 Quantitative Health Sciences, Cleveland Clinic, 9500 Euclid Ave, J1-5, Cleveland, OH 44195 USA; 4 Case Western University, 10900 Euclid Ave, Cleveland, OH 44106-7017 USA; 5 Heart and Vascular Institute, Cleveland Clinic, 9500 Euclid Ave, J1-5, Cleveland, OH 44195 USA; 6 Sanger Heart & Vascular Institute, Atrium Health, 1237 Harding Place, MOB1 Suite 5000, Charlotte, NC 28204 USA; 7 Imaging Institute, Cleveland Clinic, 9500 Euclid Ave, J1-5, Cleveland, OH 44195 USA

**Keywords:** Sex, Aortic regurgitation, Echocardiography, Cardiac magnetic resonance imaging, Echocardiography

## Abstract

**Background:**

Significant aortic regurgitation (AR) leads to left ventricular (LV) remodeling; however, little data exist regarding sex-based differences in LV remodeling in this setting. We sought to compare LV remodeling and AR severity, assessed by echocardiography and cardiovascular magnetic resonance (CMR), to discern sex-based differences.

**Methods:**

Patients with ≥ moderate chronic AR by echocardiography who underwent CMR within 90 days between December 2005 and October 2015 were included. Nonlinear regression models were built to assess the effect of AR regurgitant fraction (RF) on LV remodeling. A generalized linear model and Bland Altman analyses were constructed to evaluate differences between CMR and echocardiography. Referral for surgical intervention based on symptoms and LV remodeling was evaluated.

**Results:**

Of the 243 patients (48.3 ± 16.6 years, 58 (24%) female), 119 (49%) underwent surgical intervention with a primary indication of severe AR, 97 (82%) men, 22 (18%) women. Significant sex differences in LV remodeling emerged on CMR. Women demonstrated significantly smaller LV end-diastolic volume index (LVEDVI) (96.8 ml/m^2^ vs 125.6 ml/m^2^, p < 0.001), LV end-systolic volume index (LVESVI) (41.1 vs 54.5 ml/m^2^, p < 0.001), blunted LV dilation in the setting of increasing AR severity (LVEDVI p value < 0.001, LVESVI p value 0.011), and LV length indexed (8.32 vs 9.69 cm, p < 0.001). On Bland Altman analysis, a significant interaction with sex and LV diameters was evident, demonstrating a significant increase in the difference between CMR and echocardiography measurements as the LV enlarged in women: LVEDVI (p = 0.006), LVESVI (p < 0.001), such that echocardiographic measurements increasingly underestimated LV diameters in women as the LV enlarged. LV length was higher for males with a linear effect from RF (p < 0.001), with LV length increasing at a higher rate with increasing RF for males compared to females (two-way interaction with sex p = 0.005). Sphericity volume index was higher for men after adjusting for a relative wall thickness (p = 0.033).

**Conclusions:**

CMR assessment of chronic AR revealed significant sex differences in LV remodeling and significant echocardiographic underestimation of LV dilation, particularly in women. Defining optimal sex-based CMR thresholds for surgical referral should be further developed.

*Trial registration*: NA.

**Supplementary Information:**

The online version contains supplementary material available at 10.1186/s12968-022-00845-5.

## Introduction

Chronic aortic regurgitation (AR) results in left ventricular (LV) remodeling; however, the extent of LV remodeling in patients with hemodynamically significant AR is not uniform and it is unknown as to how variation in LV remodeling may contribute to the development of symptoms [1, 2]. Current guideline recommendations for aortic valve surgery in the setting of chronic severe AR include: symptoms, and echocardiographic thresholds for LV dilation and reduced LV ejection fraction (LVEF) [3, 4]. However, discerning severity of AR can be difficult by echocardiography alone, particularly as echocardiographic classification is associated with significant inter-observer variability [5].

Much controversy still surrounds what defines AR severity, optimal timing of intervention in the asymptomatic population, and the ideal imaging modality for AR assessment. Moreover, prior studies have demonstrated that women tend to be more symptomatic, and fewer women with severe AR are referred for surgical intervention based on LV remodeling [6, 7]. Currently, there are a paucity of data regarding sex-based differences in LV remodeling in women with significant chronic AR. While prior echocardiographic studies have not revealed significant sex-based differences, these studies may have been underpowered to discern such differences in LV remodeling due to variability in echocardiographic measurements, as well as the small proportion of women in these studies [1, 8–10]. Therefore, it is unknown if limitations in echocardiographic measurements accuracy of LV remodeling have obscured sex-based differences. Recently the evaluation of LV remodeling by cardiovascular magnetic resonance (CMR) has been shown to add incremental prognostic value to conventional clinical and echocardiographic variables, while providing comprehensive AR quantification [2, 11–13]. Thus, we hypothesized that the superior reproducibility and accuracy of CMR quantification of LV remodeling, as well as smaller coefficients of variation in measurements by CMR compared to echocardiography [14], may provide the ability to discern sex-based differences in LV remodeling in response to significant chronic AR.

We sought to evaluate the hypothesis that CMR quantification of AR severity and LV remodeling in patients with chronic AR can more optimally identify sex based differences in LV remodeling than echocardiography alone. Our study objectives were as follows: (1) to compare the correlation between LV remodeling and AR severity by echocardiography and CMR to discern sex-based differences; (2) to assess sex-based differences in LV remodeling; and (3) to assess sex-based differences in referral for surgical aortic valve intervention.

## Methods

### Study population

This is a single center retrospective cohort study of consecutive patients with at least moderate chronic AR by echocardiography, who underwent CMR within 90 days of the qualifying echocardiogram between December 1, 2005 and December 3, 2019. Patients with an LVEF < 50%, acute AR, concomitant valvular lesions moderate or greater in severity, type A dissection or aortic valve endocarditis, congenital heart disease, prior cardiac surgery, hypertrophic cardiomyopathy, cardiac amyloidosis/sarcoidosis, pericardial disease, radiation heart disease, pulmonary hypertension, restrictive/dilated/ischemic cardiomyopathy were excluded. Patients with inadequate views on echocardiography for assessment of AR or LV dimensions/volume were excluded. Clinical data were entered into the electronic medical record prospectively and extracted for analysis. This study was approved by our Institutional Review Board and granted a waiver for informed consent.

### Echocardiography

**Fig. 1:**
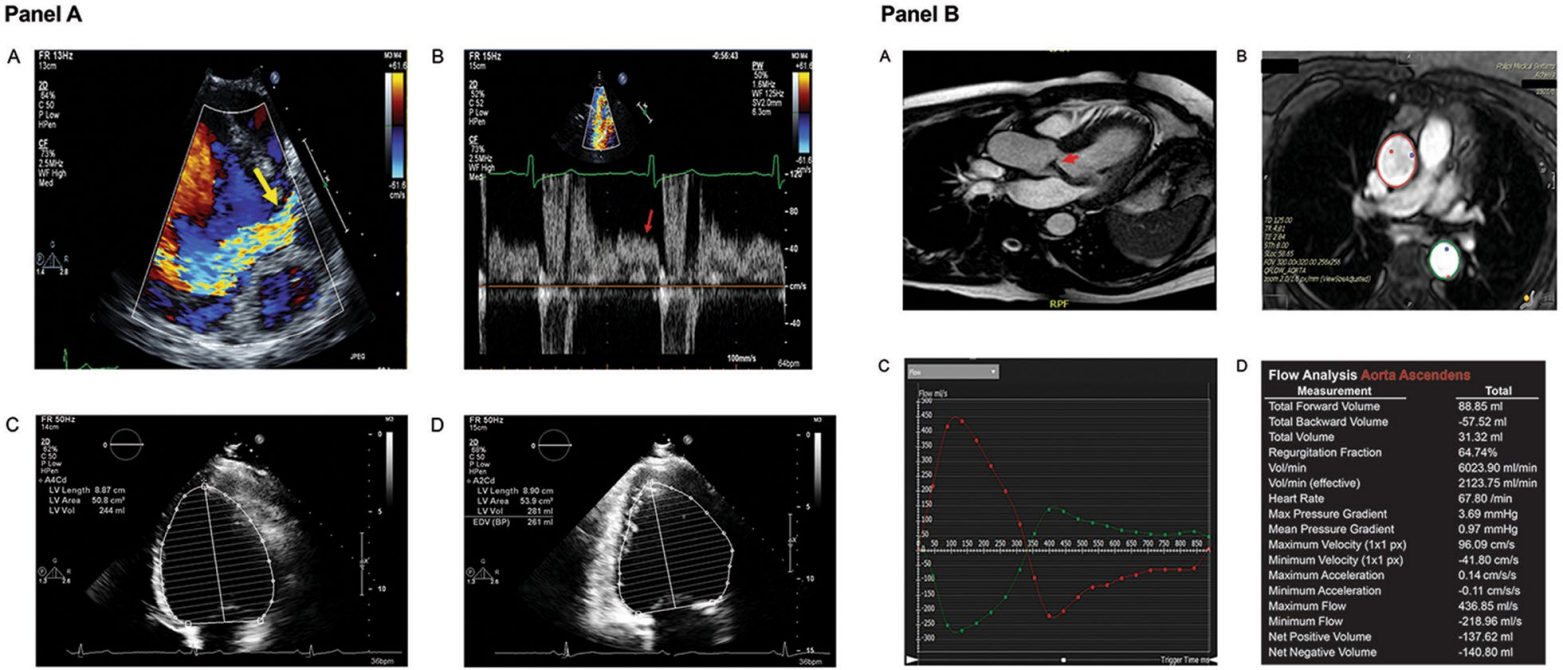
**a** Assessment of aortic regurgitation (AR) by transthoracic echocardiogram. **A** Apical 5-chamber view with Color Doppler demonstrating AR (yellow arrow). **B** Pulse-wave Doppler within the descending aorta with holodiastolic flow reversal (red arrow). **C**, **D** Apical 4-chamber and 2-chamber views (respectively) demonstrating traced left ventricular (LV) end-diastolic volume. **b** Assessment of AR by cardiovascular magnetic resonance (CMR). **A** Cine balanced steady-state free precession image demonstrating dephasing consistent with AR (red arrow). **B** Phase-contrast imaging with contours traced for the ascending (red contour) and descending (green contour) aorta. **C** Measured anterograde and retrograde flow of the ascending (red line) and descending (green line) aorta with holodiastolic flow reversal in the descending aorta (green arrows). **D** Measurements obtained from flow analysis

Comprehensive echocardiographic examination was performed for all patients using commercially available machines (Phillips Healthcare, Best, the Netherlands; Siemens Healthineers, Erlangen, Germany; or General Electric Healthcare, Chicago, Illinois, USA). LV dimensions (wall thickness, volumes and mass) were measured and calculated based on the 2015 American Society of Echocardiography (ASE) guidelines [15]. Cardiac chamber sizes were indexed to both body surface area (BSA) and height [16]. AR severity rating was stratified as mild, moderate, or severe in accordance with the ASE guidelines [17]. Additionally a hierarchical, multi-parametric method was employed, as part of the extensive efforts within our echocardiographic laboratory to decrease interobserver variability [5] (Fig. 1a).

### Cardiovascular magnetic resonance

CMR was performed based on institutional protocols (1.5 T Avanto, Siemens Healthineers, Erlangen, Germany, from 2005 to 2008, or 1.5 T Achieva or 3 T Ingenia (Phillips Healthcare, Best, the Nethernands, from 2009 to 2015). Scan parameters for balanced steady state (bSSFP) imaging on the 1.5 T scanner are as follows: TR 2.7 ms, TE 1.4 ms, acquired matrix 180 × 183, flip angle 70°, temporal resolution 30 ms. Phase contrast imaging parameters for flow quantification on the 1.5 T scanner are as follows: TR 4.8 ms, TE 2.9 ms, acquired matrix 128 × 120, flip angle 12°, temporal resolution 39 ms. Scan parameters for the 3 T scanner are as follows: bSSFP imaging: TR 2.8 ms, TE 1.4 ms, acquired matrix 192 × 177, flip angle 45°, temporal resolution 53 ms. Phase contrast imaging parameters for flow quantification on the 3 T scanner are as follows: TR 4.7 ms, TE 2.7 ms, acquired matrix 128 × 110, flip angle 12°, temporal resolution 47 ms. Epicardial and endocardial borders were traced at end-diastole and end-systole, excluding the papillary muscles, from the short axis stack bSSFP cine images to determine LV volumes and LVEF. Sphericity index was calculated as the ratio of LV end-diastolic volume to the volume of a sphere with diameter equal to the LV length measured at end-diastole in the 4 chamber view, with values closer to 1 indicating greater sphericity. Relative wall thickness (RWT) was calculated as (septal wall thickness + posterior wall thickness)/LV end-diastolic dimension) [18]. Flow quantification of AR was assessed utilizing phase contrast velocity encoded imaging acquired at the mid-ascending aorta at the level of the right pulmonary artery. This was based on prior investigations demonstrating no significant difference in regurgitant volume (RV) and fraction (RF) performed at the sinotubular junction and mid ascending aorta in a cohort which included ~ 40% non-tricuspid aortic valves and eccentric AR [19]. The contour of the aorta was traced on phase images throughout the cardiac cycle to measure antegrade and retrograde flow, which were then used to calculate RV and RF. At the time of image acquisition our lab defined severity of AR as the following: mild as a RF =< 20%, moderate as a RF of 21–39%, and severe as a RF >= 40% [20, 21]. Phase-contrast flow data and LV mass and volumes were measured, as previously described [21]. Post-processing analysis was performed with cvi42 (Circle Cardiovascular Imaging, Calgary, Alberta, Canada). The presence of holodiastolic flow reversal (HFR) in the descending thoracic aorta was also recorded and defined as persistent flow reversal ≥ 10 ml/s throughout diastole [22] (Fig. 1b).

### Follow Up

Clinical outcomes of aortic valve surgery (repair or replacement), and death were assessed for all patients until September 30, 2018. Outcomes were assessed by review of electronic medical records, or by phone call for patients not seen within our system in the prior three months using a script approved by our Institutional Review Board with two attempts at contact. Patients lost to follow up were censored at the time of last known contact.

### Statistical analysis

Continuous variables are expressed as mean ± standard deviation and categorical variables are expressed as percent. Differences in LV volumes indexed by both BSA and height, based on aortic valve morphology and sex. Non-linear regression models were built to assess the effect of RF on LV dimensions and volumes based on sex. Bland–Altman plots were constructed to assess differences in measurements between echocardiography and CMR by sex. A multivariable linear regression model was constructed to determine independent predictors of LV remodeling. Linear and nonlinear effects were considered for each continuous predictor and two-way interactions with gender were included. Sensitivity/specificity, and Youden’s index, were reported for various cut-points of CMR predictors. Statistical analyses were performed using SAS (version 9.4, SAS Institute, Cary, NC, USA). Significance was based on a p-value of < 0.05.

## Results

Two hundred forty-three patients (48.3 ± 16.6 years, 58 (24%) female patients) who had at least moderate AR on echocardiography were included in our study. Clinical demographic and imaging variables based on gender are described in Table 1. Cardiac measurements were indexed by BSA as well as by height. The prevalence of a bicuspid aortic valve was higher in men (71% vs. 35%, p value < 0.001). Women were more symptomatic (p = 0.006), in the setting of smaller LV volumes (p < 0.001) when indexed by both BSA and height, with similar categorization of AR severity by echocardiography (Table 2), but smaller mean RF by CMR (Table 1). CMR assessed LV diameters, indexed by height, were significantly larger in women; though this difference was not significant when indexed by BSA. Echocardiographic and CMR measurements according to aortic valve morphology, indexed by BSA and height are listed in Additional file 1: Supplementary Tables 1 and 2, respectively.

**Table 1:** Baseline clinical, echocardiographic and CMR data

	Male (n = 185)	Female (n = 58)	Total (n = 243)	p-value
Clinical data				
Age (years)	46.8 ± 16.5	53.2 ± 16.2	48.3 ± 16.6	0.017
Heart rate (beats per minute)	67 ± 12	75 ± 12	69 ± 13	< 0.001
Systolic blood pressure (mmHg)	129 ± 18	128 ± 21	129 ± 19	0.439
Diastolic blood pressure (mmHg)	68 ± 12	69 ± 12	68 ± 12	0.576
Body mass index	27.7 ± 5.1	28.2 ± 6.6	27.8 ± 5.5	0.635
Height (cm)	178.5 ± 7.5	163.5 ± 6.4	175.2 ± 9.5	< 0.001
Type of aortic valve				
Unicuspid Bicuspid Tricuspid Quadricuspid	2 (1.1%) 128 (70.7%) 50 (27.6%) 0	0 20 (34.5%) 37 (63.8%) 1 (1.7%)	2 (0.8%) 148 (61.9%) 87 (36.4%) 1 (0.4%)	< 0.001
Dilated ascending aorta (> 4 cm)	74 (43.3%)	27 (49.1%)	101 (44.7%)	0.451
Coronary artery disease	17 (9.2%)	8 (13.8%)	25 (10.3%)	0.314
Atrial fibrillation	16 (8.7%)	1 (1.7%)	17 (7.0%)	0.071
Chronic kidney disease	6 (3.2%)	4 (6.9%)	10 (4.1%)	0.222
Diabetes mellitus	9 (4.9%)	1 (1.7%)	10 (4.1%)	0.293
Hypertension	94 (50.8%)	34 (58.6%)	128 (52.7%)	0.299
NYHA class:				
1 2 3	139 (75.1%) 38 (20.5%) 7 (3.8%)	29 (50.0%) 23 (39.7%) 6 (10.3%)	168 (69.1%) 61 (25.1%) 13 (5.4%)	0.003
Echocardiographic data indexed to BSA				
LVEDVI (ml/m^2^)	87.0 ± 31.5	60.1 ± 24.0	81.0 ± 32.0	< 0.001
LVESVI (ml/m^2^)	35.9 ± 18.4	25.0 ± 14.2	33.5 ± 18.1	< 0.001
LVEDDI (cm/m^2^)	2.8 ± 0.57	2.6 ± 0.44	2.7 ± 0.50	0.041
LVESDI (cm/m^2^)	1.9 ± 0.5	1.7 ± 0.3	1.8 ± 0.4	0.024
LV mass index (g/m^2^)	133.9 ± 47.0	108.5 ± 39.1	128.3 ± 46.5	< 0.001
LVEF (%)	57.9 ± 6.5	58.6 ± 6.5	58.0 ± 6.5	0.374
CMR data indexed to BSA				
LVEDVI (ml/m^2^)	125.6 ± 39.4	96.8 ± 27.0	119.2 ± 38.9	< 0.001
LVESVI (ml/m^2^)	54.5 ± 23.8	41.1 ± 16.4	51.5 ± 23.0	< 0.001
LVEDDI (cm/m^2^)	2.9 ± 0.54	2.8 ± 0.51	2.9 ± 0.53	0.520
LVESDI (cm/m^2^)	1.9 ± 0.5	1.8 ± 0.5	1.9 ± 0.5	0.300
Length	9.7 ± 1.1	8.3 ± 0.7	9.4 ± 1.2	< 0.001
LV mass index (g/m^2^)	78.3 ± 27.1	66.2 ± 22.4	75.7 ± 26.6	< 0.001
LVEF (%)	57.5 ± 6.5	58.3 ± 6.9	57.7 ± 6.6	0.733
RF	28.1 ± 15.5	21.2 ± 17.5	26.4 ± 16.2	0.001
RV	38.9 + 29.8	18.0 ± 16.4	34.0 ± 28.6	< 0.001
HFR	35.3%	27.0%	33.7%	0.439
Echocardiographic data—indexed to height				
LVEDVI (ml/m^2.7^)	37.8 ± 13.7	28.8 ± 11.6	35.8 ± 13.8	< 0.001
LVESVI (ml/m^2.7^)	15.8 ± 7.9	11.9 ± 6.8	15.0 ± 7.8	< 0.001
LVEDDI (cm/m^2.7^)	1.2 ± 0.22	1.3 ± 0.19	1.2 ± 0.22	0.009
LVESDI (cm/m^2.7^)	0.78 ± 0.17	0.81 ± 0.14	0.79 ± 0.16	0.131
LV mass index (g/m^2.7^)	58.4 ± 20.5	53.7 ± 19.9	57.4 ± 20.4	0.038
CMR data indexed to height				
LVEDVI (ml/m^2.7^)	53.7 ± 17.3	46.4 ± 14.1	52.0 ± 16.9	0.002
LVESVI (ml/m^2.7^)	23.2 ± 10.2	19.7 ± 8.1	22.4 ± 9.8	0.005
LVEDDI (cm/m^2.7^)	1.2 ± 0.21	1.4 ± 0.22	1.3 ± 0.22	< 0.001
LVESDI (cm/m^2.7^)	0.80 ± 0.18	0.87 ± 0.21	0.82 ± 0.19	0.019
LVMi (g/m^2.7^)	33.9 ± 12.4	32.1 ± 12.3	33.5 ± 12.4	0.231

Continuous variables are expressed as mean ± standard deviation and categorical variables are expressed as number (percent)

*BSA* body surface area, *CMR* cardiovascular magnetic resonance, *HFR* holodiastolic flow reversal, *NYHA* New York Heart Association, *LVEDDI* left ventricular end-diastolic dimension index, *LVEDVI* left ventricular end-diastolic volume index, *LVESDI* left ventricular end-systolic dimension index, *LVESVI* left ventricular end-systolic volume index, *LV* left lentricle/left ventricular mass index, *LVEF* left ventricular mass index, *RF* regurgitant fraction, *RV* regurgitant volume

**Table 2:** Comparison of severity of aortic regurgitation by echocardiography and CMR

		AR Severity by echocardiography	
		Moderate	Severe
AR severity by CMR (all)	Mild	91	5
	Moderate	**61**	29
	Severe	19	**38**
AR severity by CMR (males)	Mild	61	3
	Moderate	**50**	23
	Severe	16	**32**
AR severity by CMR (females)	Mild	30	2
	Moderate	**11**	6
	Severe	3	**6**

Bolded values represent cases with concordant severity on both echocardiography and CMR

*AR* aortic regurgitation, *AR* aortic regurgitation, *CMR* cardiovascular magnetic resonance imaging

The severity of AR by echocardiography and CMR was concordant in 41% of cases; in 51% of cases AR severity was judged to be more severe by echocardiography and in 8% more severe by CMR. In female patients the two modalities agreed in 29% of cases, a significantly smaller proportion of cases, and was categorized as higher severity by CMR as compared with echocardiography in 5%. In male patients the two modalities agreed in 44%, significantly more than for females even after adjusting for valve type (p = 0.017), with 9% of men measured as having more severe AR on CMR than echocardiography (Table 2).

### Comparison of echocardiographic and CMR measurement of the impact of AR on left ventricular size

#### Left ventricular volumes

**Fig. 2:**
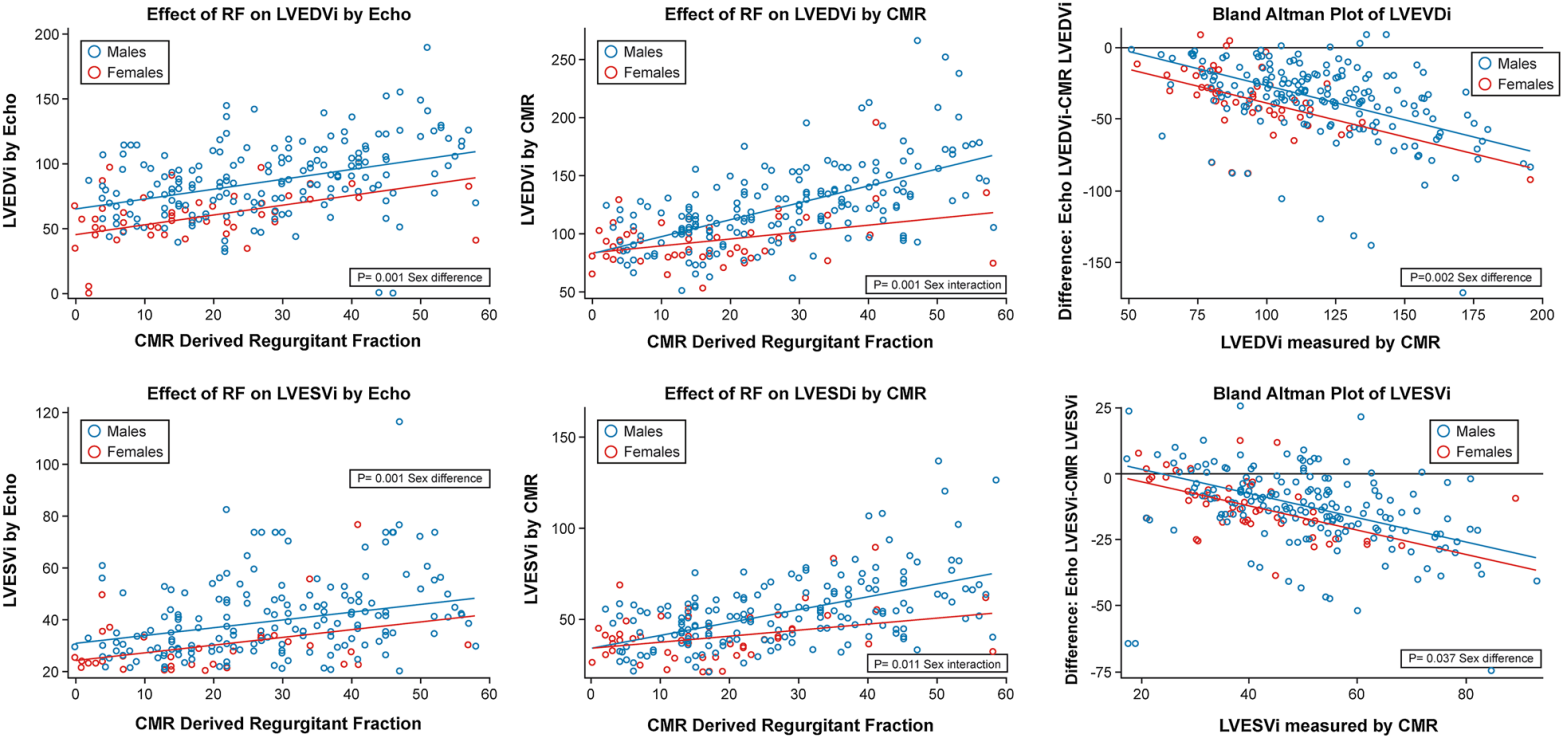
Distribution of left ventricular end-diastolic volume index (LVEDVI) and left ventricular end-systolic volume index (LVESVI) by echocardiography and CMR and severity of aortic regurgitation. Top Row (left to right). LVEDVI by echocardiography to severity of AR measured by CMR derived aortic regurgitant fraction by sex; LVEDVI by CMR to severity of AR measured by CMR derived aortic regurgitant fraction by sex. Bland Altman analysis comparing LVEDVI measured by CMR vs echocardiography by sex. Bottom Row (left to right). LVESVI by echocardiography to severity of AR measured by CMR derived aortic regurgitant fraction by sex; LVESVI by CMR to severity of AR measured by CMR derived aortic regurgitant fraction by sex. Bland Altman analysis comparing LVESVI measured by CMR vs echocardiography by sex. Black line denotes no difference between modalities

With increasing RF, both LV end-diastolic volume index (LVEDVI) and LV end-systolic volume index (LVESVI) by echocardiography and CMR increased (p < 0.001, all) (Fig. 2). LVEDVI measured by CMR was an average of 38.4 ml/m^2^ larger than by echocardiography (p < 0.001, 95% CI of [34.8, 41.9]), and this difference in measurement of LVEDVI between CMR and echocardiography was larger for women (p = 0.002). Multivariable models were created to determine the impact of sex on LVEDVI and LVESVI. The final models for LVEDVI by echocardiography and CMR included sex, age, heart rate, RF, and aortic valve type. Sex was a significant independent predictor of LVEDVI in both models, and men demonstrated significantly larger LVEDVI by CMR particularly in the presence of higher RF. Furthermore, significant sex interaction with CMR derived LVEDVI (p < 0.001) and LVESVI (p = 0.001) with increasing RF, such that women demonstrated a more blunted increase LV volumes in the setting of increasing RF. In regards to echocardiographic measurements, LVEDVI measured by echocardiography demonstrated the same significant sex difference (p = 0.001, both). However, the sex interactions with LV volumes and RF were not present with echocardiographic measures of LV volumes. The final models for LVESVI by echocardiography and CMR included age and aortic valve type. Sex was a significant independent predictor of LVEDVI in both models, with men again demonstrating significantly larger LVESVI by CMR with increasing RF (p = 0.011), with similar findings when LVESVI was measured by echocardiography (p = 0.026).

#### Left ventricular diameters

**Fig. 3:**
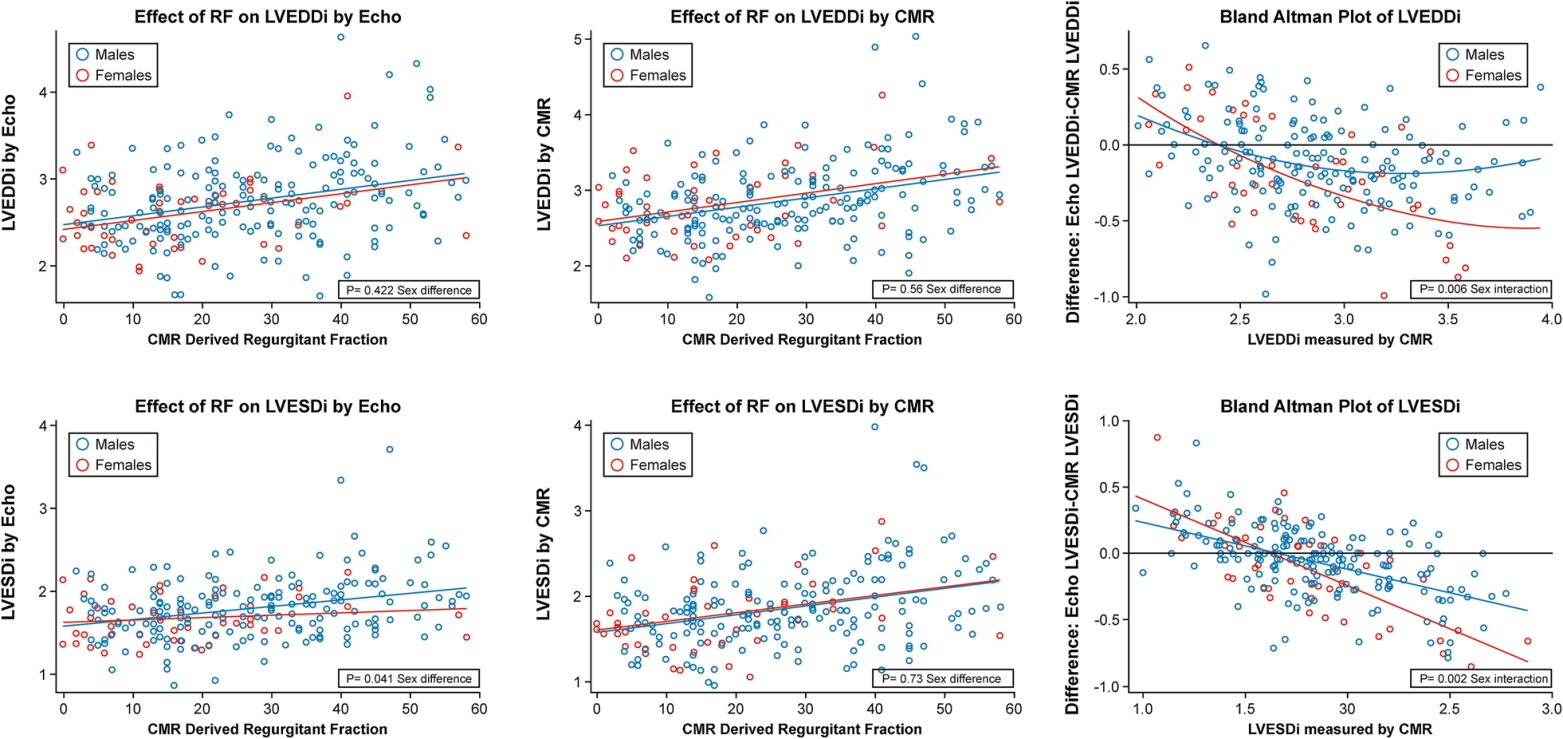
Distribution of left ventricular end-diastolic dimension index (LVEDDI) and left ventricular end-systolic dimension index (LVESDI) by echocardiogram and CMR and severity of aortic regurgitation. Top Row (left to right). Comparison of LVEDDI by echocardiography to severity of AR measured by CMR derived aortic regurgitant fraction by sex. Comparison of LVEDDI by CMR to severity of AR measured by CMR derived aortic regurgitant fraction by sex. Bland Altman analysis comparing LVEDDI measured by CMR vs echocardiography by sex. Bottom Row (left to right). Comparison of LVESDI by echocardiography to severity of AR measured by CMR derived aortic regurgitant fraction by sex. Comparison of LVESDI by CMR to severity of AR measured by CMR derived aortic regurgitant fraction by sex. Bland Altman analysis comparing LVESDI measured by CMR vs echocardiography by sex. Black line denotes no difference between modalities

Both LV end-diastolic dimension index (LVEDDI) and LV end-systolic dimension index (LVESDI) by echocardiography and CMR increased with increasing RF (p < 0.001, all) (Fig. 3). Multivariable models were again created to determine the impact of sex on LVEDDI and LVESDI measured by echocardiography and CMR. After adjusting for age, blood pressure and aortic valve morphology there were no sex differences identified when LVEDDI was measured by echocardiography (p = 0.422). On unadjusted analysis, women demonstrated similar LVEDDI, despite significantly smaller LVEDVI, compared to men. However, after adjusting for age, blood pressure, and aortic valve morphology, sex emerged as a significant independent predictor of LVEDDI when it was measured by CMR, with significantly higher LVEDDI values for females (p = 0.008). On Bland Altman analysis, there was also a significant interaction between sex and the magnitude of the difference in LVEDDI by echocardiography and CMR, such that as LVEDDI increased the magnitude of difference between LVEDDI measurements by echocardiography versus CMR increased more so for females with larger LVEDDI, than it did for males (p = 0.006). Table 3 outlines sex base differences in LVEDDI as LVEDDI increased. For example, women with LVEDDI > 3.0 cm/m^2^ demonstrated 2 × the mean difference between LVEDDI measured by CMR vs echocardiography seen in men (Table 3). After adjusting for age, LVESDI women again demonstrated similar LVESDI compared to men, despite significantly smaller LVESVI, compared to men. Interestingly, echocardiography was able to accurately identify increasing LVESDI measurements in men, when compared to CMR measurements. However, echocardiography did not accurately identify increasing LVESDI measurements in women, when compared to CMR, thus resulting in significant sex differences in LVESDI measurements by echocardiography (p = 0.041). As a result, echocardiography demonstrated decreased sensitivity to identify increasing LVESDI with increasing RF in women, compared to CMR (Fig. 3). The magnitude of the difference in LVESDI by echocardiography and CMR increased, particularly for women, with increasing LVESDI (p < 0.001). Bland Altman analysis demonstrates significant sex differences in increasing LVESDI measurement error by echocardiography as LVESDI increased (p = 0.002, sex interaction) (Fig. 3). Table 4 outlines sex based differences in LVESDI as LVESDI increased. For example, women with LVESDI > 2.5 cm/m^2^ demonstrated > 2 × the mean difference between LVESDI measured by CMR vs echocardiography seen in men (Table 4).

**Table 3:** Mean differences in LVEDDI between CMR and echocardiography based on sex

LVEDDI by CMR	Male (n = 185)	Female (n = 58)	p-value
< 3.0	− 0.0522 (0.270)	− 0.121 (0.292)	0.182
> 3.0	− 0.177 (0.226)	− 0.349 (0.301)	0.009

**Table 4:** Mean differences in LVESDI between CMR and echocardiography based on sex

LVESDI by CMR	Male (n = 185)	Female (n = 58)	p-value
< 1.5	0.128 (0.216)	0.161 (0.253)	0.630
1.5–2.5	− 0.111 (0.216)	− 0.138 (0.278)	0.556
2.5	− 0.3230 (0.2494)	− 0.698 (0.3538)	0.009

#### Left ventricular length/sphericity

**Fig. 4:**
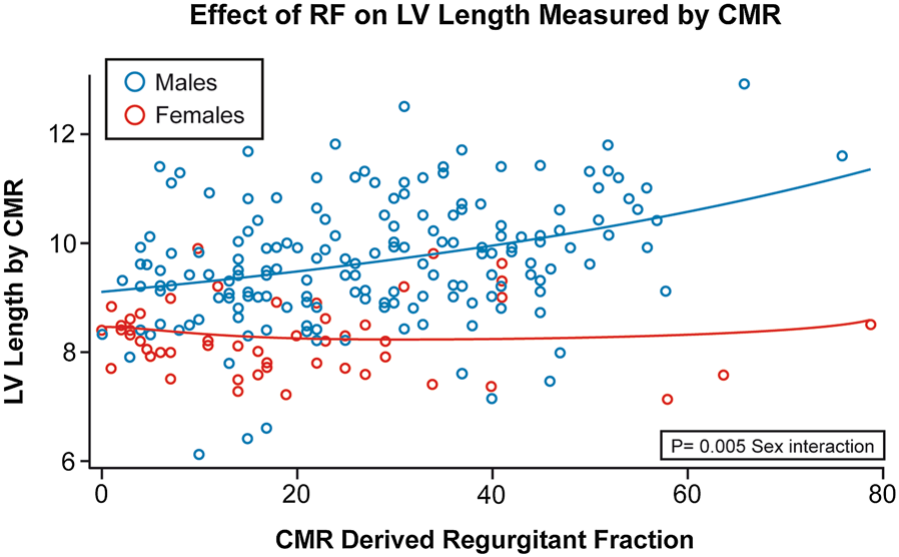
Distribution of left ventricular length by CMR by severity of aortic regurgitation

**Fig. 5:**
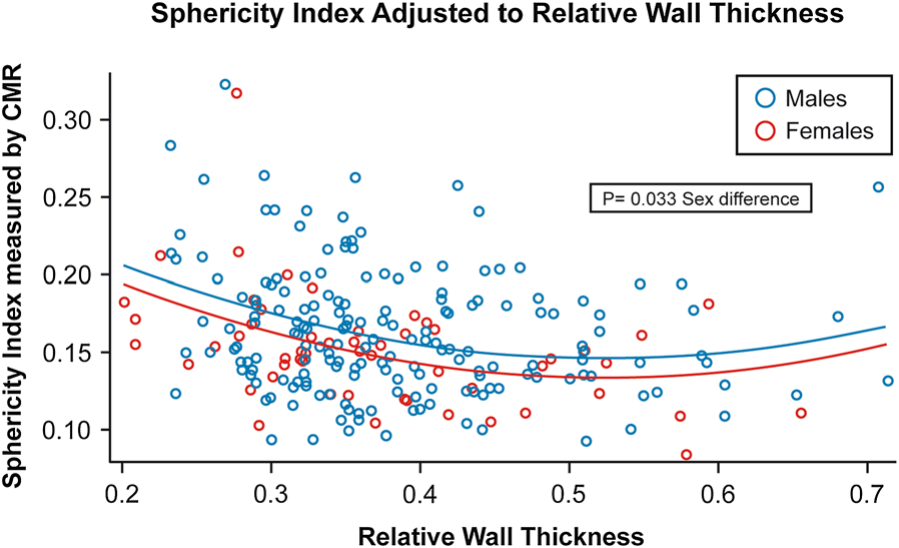
Distribution of Sphericity Volume Index adjusted to Relative Wall Thickness

LV length, sphericity, and RWT were measured to describe LV shape to further explore sex differences LV volumes, in the setting of similar LV diameters. LV length was longer for males (p < 0.001) and increased in a linear fashion with increasing RF in both male and female patients, (p < 0.001). However, LV length increased at a higher rate for males, as RF increased (two-way interaction with sex p = 0.005) (Fig. 4). LV sphericity volume index was calculated and adjusted for regional wall thickness to account for both LV length, diameter, and volume, as outlined by Nakamori et al. [18] Men demonstrated significantly higher sphericity volume index, compared to women (p = 0.033), and sphericity volume index decreased non-linearly as RWT increased for both sexes (p = 0.003) (see Fig. 5).

#### Association of HFR with RF

Given the recent demonstration of HFR defining significant AR [11, 13], a univariate analysis was performed to predict the presence of HFR on CMR. While RF, RV, and LVEDVI were significant predictors (p < 0.001, all), sex was not a significant predictor of the presence of HFR (p = 0.144). Sensitivity/specificity analysis demonstrated that an RF > 30% provided the excellent discrimination for predicting the presence of HFR (Youden’s index 0.827), with sensitivity = 1.0 and specificity = 0.827).

### Surgical referral

During the follow up period, 156 patients (121 men and 35 women) underwent aortic valve surgery. Severe AR was the primary indication for aortic valve surgery in 119 patients (97 men and 22 women). Mean time from the CMR to date of surgery was 321 days, 62.2% within 6 months, with no difference between men and women. Adjusting for age, HR, RF, and aortic valve type, women who underwent aortic valve surgery with a primary indication of severe AR had significantly smaller LVEDVI (women: 91.3 ± 27.6 ml/m^2^ vs men: 143.2 ± 47.6 ml/m^2^, p = 0.003), LVESVI (women: 37.8 ± 14.1 ml/m^2^ vs men: 64.1 ± 30.4 ml/m^2^, p = 0.041), and LV length (women: 8.2 ± 0.8 mm vs men: 9.9 ± 1.2 mm, p = 0.001), but LVEDDI and LVESDI by CMR were similar: women: 2.9 ± 0.4 cm/m^2^ vs men: 3.1 ± 0.6 cm/m^2^, (p = 0.754), and women: 1.8 ± 0.4 cm/m^2^ vs men: 2.1 ± 0.6 cm/m^2^, (p = 0.7 and 0.48, respectively).

## Discussion

In this study, we found that CMR evaluation of chronic AR elucidated significant sex differences in LV remodeling. While women demonstrated consistently smaller LV indexed volumes in response to chronic AR, women demonstrated significantly larger indexed LV basal diameters. Given that CMR is considered the gold standard modality for LV remodeling quantification, our study suggests that echocardiographic assessment of LV remodeling becomes less accurate as the LV enlarges, for both men and women. However, echocardiographic assessment of AR severity and LV diameters is more significantly discrepant from CMR assessment, compared to men, with strikingly significant margins of error in echocardiographic measurements in women with larger indexed LV diameters.

CMR arguably provides the most comprehensive phenotypic evaluation of patients with chronic AR, providing gold standard measurements of LV remodeling, quantitative measurements of AR volume and RF, and precise evaluation of the thoracic aorta for the assessment of concomitant aortopathy. CMR is considered the gold standard for assessment of LV size and function, with impressively small coefficients of variability (2.9% for end-diastolic LV volume, 6.5% for end-systolic LV volume, 3.9% for stroke volume, and 2.8% for LV mass) which have been shown to be > 50% lower than the standard coefficients of variability acquired by two-dimensional echocardiography [14]. Despite these strengths of CMR for the assessment of LVR, there are no definitive clinical/societal guidelines for defining CMR thresholds to categorize severity of AR, or threshold of LV dilation measured by CMR included in the criteria for surgical indications for aortic valve intervention. Our study examines the association between echocardiography versus CMR quantification of AR severity and resultant LV remodeling, and further distinguishes significant sex differences in LVR in response to chronic AR. Our study demonstrates 2 main findings: (1) CMR evaluation of LVR demonstrated significant differences with echocardiography, with increasing differences in CMR vs echo measurements of LV remodeling as the LV size increases, particularly in women with LV dilation; and (2) CMR elucidated significant sex-based phenotypes of LV remodeling. Furthermore, our study demonstrated a high prevalence of symptoms in women. Additional studies are needed to determine if sex differences in phenotypic LV remodeling in response to chronic AR, may impact the development of symptoms.

### Sex differences in LV remodeling in response to chronic aortic regurgitation

Our study demonstrates significant sex-based differences in LV remodeling, and the impact of sex on the accuracy of echocardiography to quantify LV remodeling in the presence of significant chronic AR. One prior study examining differences in sex-based LV remodeling by CMR in AR also demonstrated limited compensatory LV dilation in women with chronic AR compared to men [23]. However, a significant proportion of patients within this cohort had moderate or greater aortic stenosis (27%) or mitral regurgitation (23%) confounding the ability to assess the impact of isolated AR on LV remodeling. Our current study, builds upon Kammerlander et al.’s findings, and further defines sex differences in phenotypic geometrical differences in LV remodeling and sex-based differences in LVESVDI thresholds and echocardiographic LVESDI margins of error. Given the improved accuracy of CMR quantification of LVR, CMR evaluation in our study defined a more marked increase in LVEDVI and LVESVI for both men and women in the setting of increasing AR severity, when compared to echocardiography. Additionally LV length increased with increasing AR severity, though the rate of increase was significantly higher in men. However, upon further comprehensive assessment of LV remodeling beyond LV volumes by CMR, our findings demonstrate that evaluation of LV remodeling is incompletely described by LV diameters or LV volumes in isolation. In our study, the integration of LV diameters, LV length, wall thickness, LV volumes, and LV sphericity provided a comprehensive assessment which elucidated distinct sex-based phenotypes of LV remodeling in response to chronic AR.

While previous research have also demonstrated sex-based differences in LVR in response to increased afterload, such as severe aortic stenosis, there are limited data regarding phenotypic differences in remodeling in response to AR, based on sex. Women in our study demonstrated significantly smaller CMR derived LV volumes, even when indexed to BSA. This significant difference was seen at all levels of AR severity, and women demonstrated a blunted increase in LV volumes with increasing AR severity. Furthermore, women in our study surprisingly demonstrated larger LVEDDI after adjusting for age, blood pressure, RF, and aortic valve morphology, while demonstrating significantly smaller LV length. Furthermore, when CMR measurements were indexed to height, rather than BSA, indexed LV diameters were significantly larger in women compared to men, despite having significantly smaller indexed LV volumes.

Additionally men had consistently higher sphericity volume index than women after adjusting for relative wall thickness. These findings suggests that male LV remodeling is more consistent with a spherical phenotype and female LV remodeling is more consistent with a conical phenotype.

**Fig. 6:**
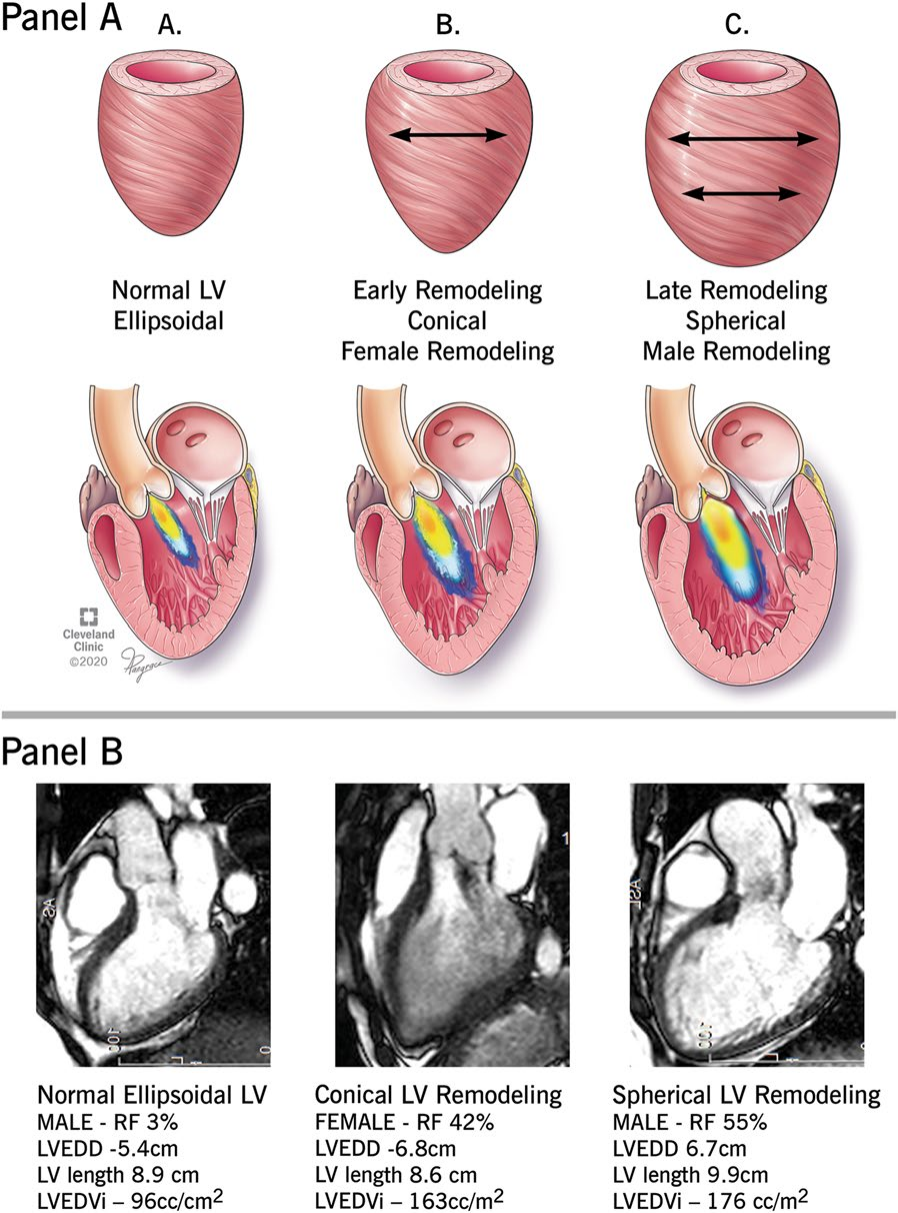
**a** Differences in LV remodeling Phenotypes based on early vs late phase of remodeling. Illustration demonstrates the differences of regional left ventricular dilation as a function of time. **b** Sex based Differences in LV Remodeling Phenotypes. CMR images demonstrate corresponding sex based differences in LV remodeling with regional left ventricular dilation in the presence of chronic severe AR

Similar differences in phenotypic geometric remodeling have previously been described in an animal model of chronic overload [24]. Ashikaga et al. demonstrated that chronic volume overload resulted in conical LV geometry in the early phase with an isolated increase in basal diameters, and that LV remodeling progressed to a more spherical geometry in the late phase, with increases in both apical and basal dimensions. Thus, our findings suggest that women may demonstrate a phenotype that is more consistent with early remodeling, with a blunted response in the ability for further dilation (Fig. 6). We postulate that this blunted compensatory response in further LV dilation may result in increased wall stress in the setting of chronic volume overload, and may explain the presence of more significant symptoms in the female patients included in our study. Further mechanistic studies are needed to determine if the blunted response to LV compensation/remodeling and differences in LV geometry in the setting of significant AR result in increased LV wall stress, diastolic dysfunction and, consequently, symptoms.

### Sex-based differences in referral for surgical intervention

**Fig. 7:**
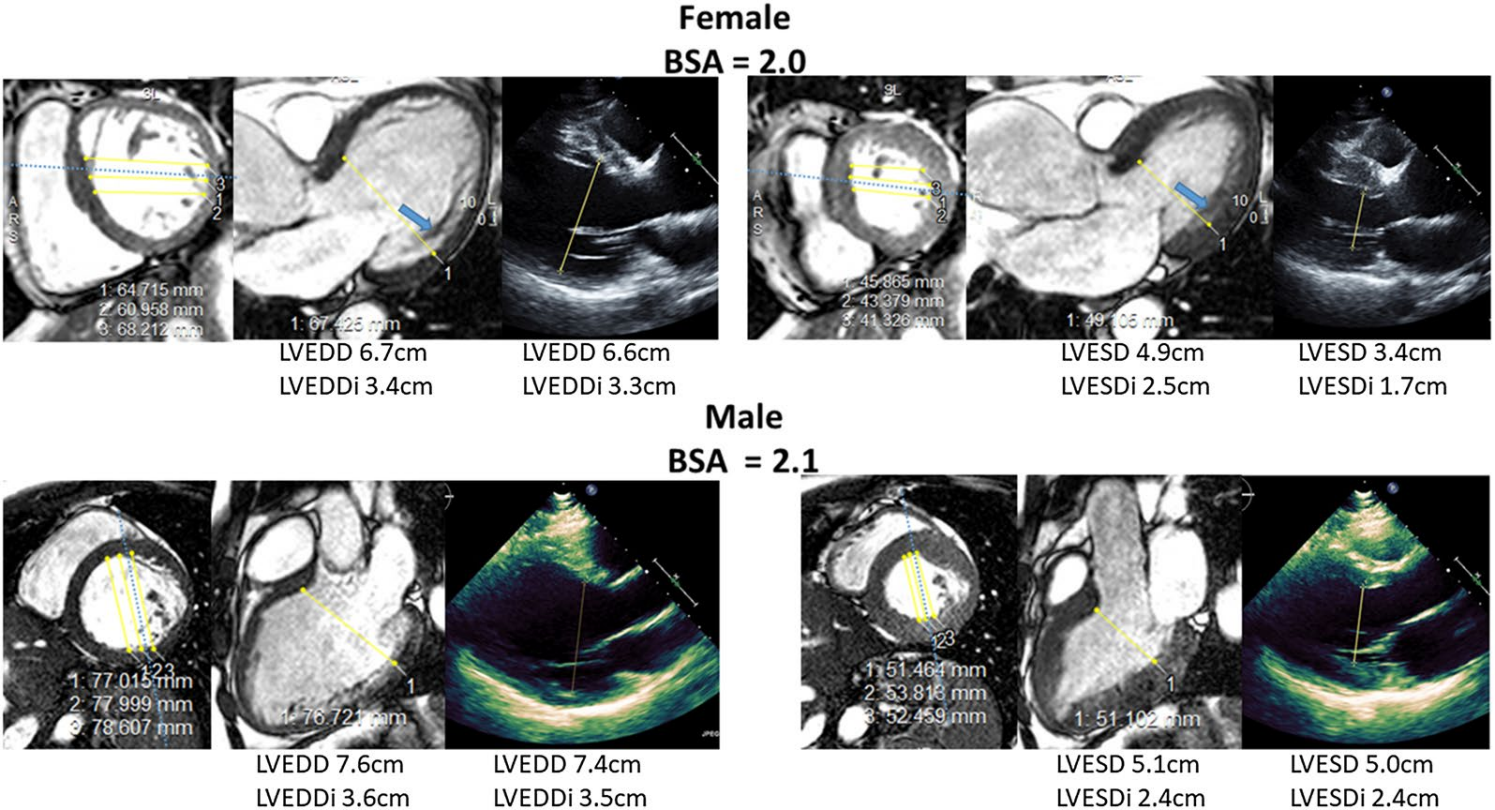
Differences in LV diameter measurements: CMR vs echocardiography. Examples of a male and female patients with severe AR and severe LV dilation to illustrate multiple etiologies for discrepant LV diameter measurements in women. Blue arrow denotes prominent trabeculations in the basal inferolateral wall. Dotted blue line denotes imaging plane that was prescribed to obtain the CMR LV outflow tract/3chamber cine image

Previous studies have demonstrated sex differences in referral patterns for surgical aortic valve intervention, such that men tend to be referred for aortic valve surgery due to a dilated LV or depressed LVEF, whereas women tend to be referred in the setting of symptoms and often at an older age than their male counterparts [6]. Our study is the first to demonstrate that women are unlikely to develop similar extent and pattern of LV dilation in response to chronic AR. The current guideline recommendations for referring asymptomatic patients with chronic AR include LVESD > 5.0 cm or LVEDD > 6.5 cm/ > 7.0 cm based on echocardiographic data [3, 25]. Recently, Nesius et al. demonstrated differences in CMR vs echocardiographic measurements of LV diameters, such that echocardiography underestimated CMR derived LVEDD and LVESD by 6.6 mm (*p* < 0.001, CI 5.8–7.7) and 5.9 mm (*p* < 0.001, CI 4.1–7.6), respectively [26]. Our data confirms these findings of systematic underestimation of LV diameters by echocardiography, but further demonstrates significant sex differences in accuracy of LV diameter based on imaging modality. In our study, differences in LV volumes and dimensions were accentuated in the setting of increasing volumes and dimensions, and there was also a significant interaction between magnitude of LV dimensions and sex such that the magnitude of the difference in measurements in LVEDDI and LVESDI between echocardiography and CMR increased more markedly for females than males with larger LV dimensions. The etiology for the increased error in echocardiographic measurements in women is likely multifactorial. Figure 7 illustrates factors that may impact accuracy of LV diameter measurements. This figure presents the comparison of the basal slice in 2 patients with severe AR and resultant severe LV dilation, and demonstrates that the female pattern of dilation resulted in an ellipsoid shape in the basal slice. As a result, potential echocardiographic acquisition of the parasternal long/LV outflow tract image (i.e. just above or below the dotted line) can result in significant variation in measurements. On the other hand, the short axis basal slice in the male patient demonstrates symmetric dilation resulting in a circular shape that is less prone to error in measurements with differing image acquisition planes. Furthermore, significant trabeculations are seen along the basal inferolateral wall (Blue arrow) in the female patient, and, the echocardiographic image fails discern the trabeculations from the compacted myocardium during systole, resulting in a significantly smaller LVESD measurement compared to CMR. Lastly, women may have a higher prevalence of less optimal acoustic windows or image quality due to breast attenuation, which may obscure the ability to definitively identify the correct endocardial border.

Our study findings have important clinical implications, as two recent studies demonstrated increase in adverse post-operative outcomes and survival for patients who underwent surgical AVR with LVESDI 2.0–2.5 and > 2.5 cm/m^2^ [27, 28]. These findings suggest that the current guideline recommendations should be updated to include LVESDI with a threshold of 2.0–2.5 cm/m^2^. Thus, underestimation of LVESDI by echocardiography may lead to delayed surgical intervention and worse outcomes. Because men demonstrated a consistent underestimation of LV diameters by echocardiography, a simple correction factor could be derived for echocardiography-derived LV diameters in men. However, the difference between LV diameters by echocardiography and CMR increased with increasing dimension for women in our study, and women with LVESDI by CMR > 2.5 cm/m^2^ demonstrated an underestimation of LVESDI by echocardiography by an average of 0.70 ± 0.35)cm/m^2^. This significant discrepancy in LVESDI measurements demonstrates the impact of sex on the accuracy of LVESDI measurements in a range that has critical importance in regard to surgical referral and association of adverse post-surgical outcomes. Our findings, in conjunction with recent CMR studies by Kammalander et al. [23] and Malahfji el al. [29] demonstrate the unique ability for CMR to more accurately characterize sex based differences in adverse LV remodeling and resultant impact on adverse outcomes, thus suggesting that CMR may be the best suited modality to derive sex-based thresholds for surgical referral in patients with significant AR. Further studies are needed to determine if the higher prevalence in symptoms at lower severity of chronic AR in women may be attributed to differences in compensatory remodeling, with resultant increased wall stress and elevated LV filling pressures. Additionally, large multi-center studies will be required to determine if this difference in compensatory LV remodeling is associated with adverse clinical outcomes and if women would benefit from alternative surgical referral criteria.

### Limitations

While this study is one of the largest comparative studies of echocardiography versus CMR in patients with chronic AR, this was a single-center retrospective cross-sectional study examining patients referred for echocardiography and CMR at the discretion of the ordering provider. Echocardiographic contrast was utilized in a minority of the studies (3.6%) and thus conclusions cannot be made regarding whether the use of endocardial border definition contrast to better define the endocardial borders for measurement of LV volumes would alter the findings of sex-based differences. Furthermore, patients with an eGFR < 30 ml/min/1.73 m^2^ and implantable cardiac devices were excluded from our study. Therefore, we cannot exclude the presence of selection bias. Because these CMR studies were clinically indicated studies, the findings of the study may have influenced the decision to refer patients for surgical intervention. This study included a relatively small number of women; however, given the superior reproducibility of CMR measurements of LV remodeling our study is adequately powered to assess differences in LVR assessed by CMR, based on previously published data [14, 30].

## Conclusions

Our study demonstrates the ability of CMR to provide accurate and comprehensive quantitative assessment of LV remodeling in the setting of chronic AR. CMR quantification of LV dilation revealed important and novel sex-based phenotypes of LV remodeling and sex differences in symptomatology in response to chronic AR. Based on our findings, CMR provides important evaluation of chronic AR, particularly in regards to the potential for determination of optimal thresholds for surgical referral, which should include sex-specific thresholds for LV remodeling. There is a critical need to derive CMR standardized thresholds for LV volumes and LV diameters, and cutoff values for categorization of AR severity, which should be also incorporated into the societal clinical guidelines for surgical referral for aortic valve intervention. Further studies are needed to determine the pathophysiology/mechanisms of blunted LV remodeling in women in the presence of chronic AR, and how these differences might impact therapeutic management and subsequent clinical outcomes.

## Supplementary Information

**Additional file 1**: **Table S1**. Echocardiographic and CMR Data Indexed by BSA and Stratified Based on Aortic Valve Morphology **Table S2**. Echocardiographic and CMR Data Indexed by Height and Stratified Based on Aortic Valve Morphology

## Data Availability

The datasets used and/or analysed during the current study are available from the corresponding author on reasonable request.

## Abbreviations

AR: Aortic regurgitation
ASE: American Society of Echocardiography
BSA: Body surface area
bSSFP: Balanced steady state free precession
CMR: Cardiovascular magnetic resonance
HFR: Holodiastolic flow reversal
LV: Left ventricle/left ventricular
LVEDDI: Left ventricular end-diastolic dimension index
LVEDVI: Left ventricular end-diastolic volume index
LVEF: Left ventricular ejection fraction
LVESDI: Left ventricular end-systolic dimension index
LVESVI: Left ventricular end-systolic volume index
RF: Regurgitant fraction
RWT: Relative wall thickness
RV: Regurgitant volume
