# Correction to: Sex-based differences in left ventricular remodeling in patients with chronic aortic regurgitation: a multi-modality study

**DOI:** 10.1186/s12968-022-00856-2

**Published:** 2022-06-28

**Authors:** Albree Tower-Rader, Isadora Sande Mathias, Nancy A. Obuchowski, Duygu Kocyigit, Yash Kumar, Eoin Donnellan, Michael Bolen, Dermot Phelan, Scott Flamm, Brian Griffin, Leslie Cho, Lars G. Svensson, Gosta Pettersson, Zoran Popovic, Deborah H. Kwon

**Affiliations:** 1 Department of Cardiology, Harvard Medical School, Massachusetts General Hospital, 55 Fruit St, Yawkey 5B, Boston, MA 02114 USA; 2 Department of Cardiology, Houston Methodist Hospital, Weill Cornell Medical College, 6565 Fannin St, Houston, TX 77030 USA; 3 Quantitative Health Sciences, Cleveland Clinic, 9500 Euclid Ave, J1-5, Cleveland, OH 44195 USA; 4 Case Western University, 10900 Euclid Ave, Cleveland, OH 44106-7017 USA; 5 Heart and Vascular Institute, Cleveland Clinic, 9500 Euclid Ave, J1-5, Cleveland, OH 44195 USA; 6 Sanger Heart & Vascular Institute, Atrium Health, 1237 Harding Place, MOB1 Suite 5000, Charlotte, NC 28204 USA; 7 Imaging Institute, Cleveland Clinic, 9500 Euclid Ave, J1-5, Cleveland, OH 44195 USA

## Correction to: Journal of Cardiovascular Magnetic Resonance (2022) 24:12 10.1186/s12968-022-00845-5

The original publication of this article was missing the affiliation of **Duygu Kocyigit**. The affiliation is available via this correction article. The original article [1] has been updated. The publisher apologizes to the authors & readers for the inconvenience.
