# Supplementary material for: Sex-based differences in left ventricular remodeling in patients with chronic aortic regurgitation: a multi-modality study
Source: J Cardiovasc Magn Reson. 2022 Feb 22;24:12. doi: 10.1186/s12968-022-00845-5 (PMC8862390; doi:10.1186/s12968-022-00845-5)
Supplement: Supplementary file 1 — Additional file 1: Table S1. Echocardiographic and CMR Data Indexed by BSA and Stratified Based on Aortic Valve Morphology Table S2. Echocardiographic and CMR Data Indexed by Height and Stratified Based on Aortic Valve Morphology [file 12968_2022_845_MOESM1_ESM.docx]

| Supplementary Table 1: Echocardiographic and CMR Data Indexed by BSA and Stratified Based on Aortic Valve Morphology | | | | |
| --- | --- | --- | --- | --- |
|  | Male  (n=185) | Female  (n=58) | Total  (n=243) | p-value |
| Echocardiographic Data Indexed by BSA  Bicuspid: |  |  |  | |
| LVEDVI (ml/m^2^) | 88.8±30.8 | 63.9±23.7 | 86.1±31.1 | <0.001 |
| LVESVI (ml/m^2^) | 38.0±18.6 | 26.7±15.6 | 36.7±18.6 | 0.002 |
| LVEDDI (cm/m^2^) | 2.8 ±0.50 | 2.8 ±0.41 | 2.8±0.49 | 0.698 |
| LVESDI (cm/m^2^) | 1.83 ±0.42 | 1.76 ±0.28 | 1.82±0.40 | 0.639 |
| LV mass index (g/m^2^) | 130.0±42 | 111.5±40 | 128.0±42 | 0.054 |
| LVEF (%) | 57.1±6.2 | 59.1±5.9 | 57.9±6.9 | 0.519 |
|  |  |  |  |  |
| CMR data indexed by BSA  Bicuspid |  |  |  | |
| LVEDVI (ml/m^2^) | 127.2±40.5 | 94.2±36.7 | 123.2±41.3 | <0.001 |
| LVESVI (ml/m^2^) | 55.5±24.8 | 40.3±19.1 | 53.6±24.6 | 0.002 |
| LVEDDI (cm/m^2^) | 2.9±0.50 | 3.0±0.47 | 2.9±0.50 | 0.505 |
| LVESDI (cm/m^2^) | 1.9±0.4 | 1.9±0.4 | 1.9±0.4 | 0.920 |
| Length | 9.75±1.05 | 8.43±0.63 | 9.59±1.10 | <0.001 |
| LV mass index (g/m^2^)  LVEF (%)  RF | 81.3±24.4  57.1±6.2  26.4±15.2 | 58.0±26.2  57.7±6.0  16.4±13.3 | 78.5±25.7  57.2±6.19  25.0±15.3 | <0.001  0.942  0.006 |
| Echocardiographic Data Indexed by BSA  Trileaflet: |  |  |  | |
| LVEDVI (ml/m^2^) | 84.9±27.7 | 57.9±23.3 | 74.5±29.1 | <0.001 |
| LVESVI (ml/m^2^) | 35.8±14.9 | 23.7±12.7 | 31.1±15.2 | <0.001 |
| LVEDDI (cm/m^2^) | 2.7 ±0.59 | 2.6 ±0.41 | 2.7±0.53 | 0.169 |
| LVESDI (cm/m^2^) | 1.8 ±0.4 | 1.7 ±0.3 | 1.7±0.4 | 0.079 |
| LV mass index (g/m^2^) | 144.2±55 | 112.1±40 | 131.8±52.2 | <0.001 |
| LVEF (%) | 57.1±5.7 | 58.8±6.7 | 57.9±6.1 | 0.123 |
|  |  |  |  |  |
| CMR data indexed by BSA  Trileaflet |  |  |  | |
| LVEDVI (ml/m^2^) | 118.5±36.3 | 97.8±27.1 | 110.1±34.3 | 0.001 |
| LVESVI (ml/m^2^) | 50.2±20.0 | 41.2±16.6 | 46.6±19.1 | 0.014 |
| LVEDDI (cm/m^2^) | 2.8±0.66 | 2.8±0.52 | 2.8±0.61 | 0.685 |
| LVESDI (cm/m^2^) | 1.9±0.6 | 1.8±0.5 | 1.8±0.6 | 0.858 |
| Length | 9.40±1.10 | 8.22±0.69 | 8.93±1.12 | <0.001 |
| LVMi (g/m^2^) | 77.4±28.2 | 70.7±23.5 | 74.7±26.5 | 0.078 |
| LVEF (%)  RF | 58.4±7.4  32.9±14.7 | 58.8±7.1  23.8±19.2 | 58.6±7.2  29.2±17.2 | 0.926  0.002 |
| Continuous variables are expressed as mean ± standard deviation and categorical variables are expressed as number (percent). NYHA = New York Heart Association; LVEDVI = left ventricular end-diastolic volume index; LVESVI = left ventricular end-systolic volume index; LVEF = left ventricular mass index. | | | | |

| Supplementary Table 2: Echocardiographic and CMR Data Indexed by Height and Stratified Based on Aortic Valve Morphology | | | | |
| --- | --- | --- | --- | --- |
|  | Male  (n=185) | Female  (n=58) | Total  (n=243) | p-value |
| Echocardiographic data indexed to height  Bicuspid: |  |  |  | |
| LVEDVI (ml/m^2.7^) | 38.3±13.7 | 29.2±10.1 | 37.3±13.6 | 0.005 |
| LVESVI (ml/m^2.7^) | 16.4±8.1 | 12.2±6.7 | 15.9±8.1 | 0.010 |
| LVEDDI (cm/m^2.7^) | 1.2 ±0.21 | 1.3 ±0.17 | 1.2±0.21 | 0.056 |
| LVESDI (cm/m^2.7^) | 0.8 ±0.2 | 0.8 ±0.1 | 0.8±0.2 | 0.364 |
| LV mass index (g/m^2.7^) | 56.2±18.8 | 51.4±18.6 | 55.7±18.8 | 0.279 |
|  |  |  |  |  |
| CMR data indexed to height  Bicuspid |  |  |  | |
| LVEDVI (ml/m^2.7^) | 54.8±17.4 | 43.4±16.2 | 53.4±17.6 | 0.003 |
| LVESVI (ml/m^2.7^) | 23.8±10.6 | 18.6±8.6 | 23.2±10.5 | 0.020 |
| LVEDDI (cm/m^2.7^) | 1.2±0.20 | 1.4 ±0.18 | 1.3±0.20 | 0.006 |
| LVESDI (cm/m^2.7^) | 0.8±0.2 | 0.9 ±0.2 | 0.8±0.2 | 0.149 |
| LV mass index (g/m^2.7^) | 35.1±11.0 | 26.8±12.0 | 34.1±11.4 | 0.008 |
| Echocardiographic data indexed to height  Trileaflet: |  |  |  | |
| LVEDVI (ml/m^2.7^) | 37.8±10.9 | 28.8±12.5 | 34.3±12.3 | <0.001 |
| LVESVI (ml/m^2.7^) | 16.1±6.3 | 11.8±7.0 | 14.4±6.9 | <0.001 |
| LVEDDI (cm/m^2.7^) | 1.2 ±0.23 | 1.3 ±0.20 | 1.2±0.22 | 0.127 |
| LVESDI (cm/m^2.7^) | 0.8 ±0.2 | 0.8 ±0.2 | 0.8±0.2 | 0.380 |
| LV mass index (g/m^2.7^) | 64.3±22.9 | 55.2±20.5 | 60.8±22.3 | 0.014 |
|  |  |  |  |  |
| CMR data indexed to height  Tricuspid |  |  |  | |
| LVEDVI (ml/m^2.7^) | 51.4±14.8 | 48.3±13.0 | 50.1±14.1 | 0.148 |
| LVESVI (ml/m^2.7^) | 21.8±8.2 | 20.3±8.0 | 21.2±8.1 | 0.251 |
| LVEDDI (cm/m^2.7^) | 1.2±0.22 | 1.4±0.24 | 1.3±0.24 | 0.001 |
| LVESDI (cm/m^2.7^) | 0.8±0.2 | 0.9±0.2 | 0.8±0.2 | 0.032 |
| LV mass index (g/m^2.7^) | 34.1±12.7 | 35.0±11.8 | 34.5±12.3 | 0.942 |
| Continuous variables are expressed as mean ± standard deviation and categorical variables are expressed as number (percent). NYHA = New York Heart Association; LVEDVI = left ventricular end-diastolic volume index; LVESVI = left ventricular end-systolic volume index; LVEF = left ventricular mass index. | | | | |
